# Population development and landscape preference of reintroduced wild ungulates: successful rewilding in Southern Italy

**DOI:** 10.7717/peerj.14492

**Published:** 2022-12-13

**Authors:** Eleonora Rivieccio, Claudia Troiano, Simona Petrelli, Valeria Maselli, Gabriele de Filippo, Domenico Fulgione, Maria Buglione

**Affiliations:** 1 Department of Humanities, University of Naples Federico II, Naples, Italy; 2 Department of Biology, University of Naples Federico II, Naples, Italy; 3 Istituto Zooprofilattico Sperimentale del Mezzogiorno, Naples, Italy; 4 Istituto Di Gestione Della Fauna, Naples, Italy

**Keywords:** Deer, Reintroduction, Landscape, Apennine

## Abstract

**Background:**

In the past decades, the abandonment of traditional land use practices has determined landscape changes inducing reforestation dynamics. This phenomenon can be contrasted with rewilding practices, *i.e.*, the reintroduction of animals that may promote the recovery of landscape diversity. In this study, we explore the dynamics of expansion of two reintroduced populations of wild ungulates, Italian roe deer (*Capreolus capreolus italicus*) and red deer (*Cervus elaphus*), assessing their contribution in the recovery of landscape diversity.

**Methods:**

By using direct and indirect information on the two species, collected by nocturnal and diurnal surveys and camera trapping, we modelled a habitat suitability map, and estimated the density and distribution of the populations. We also performed a land use changes analysis, combining the presence of wild ungulates and livestock.

**Results and Discussion:**

We demonstrated that deer dispersed gradually from their release location, increasing in population size, and this occurred in the entire study area. Moreover, we show that areas with lower grazing density are significantly affected by forest encroachment. A possible interpretation of this result could be that wild grazers (roe deer and red deer) prefer semi-open areas surrounded by the forest. This, in association with other factors, such as domestic grazing, could be one of the main responsible in maintaining landscape mosaic typical of the Apennine mountain, confirming the value of grazers as a landscape management tool. Moreover, we show the possibility to conserve through reintroduction the vulnerable *C.c. italicus*.

## Introduction

In the past decades in Southern Europe, socio-economic demands have led to the abandonment of traditional land use practices and the migration of human populations towards urban and industrial areas (Colantoni et al., 2017), especially in mountain landscapes and in the Mediterranean hinterland (Plieninger et al., 2014). The disappearance of these practices entails a number of consequences for ecosystem functionality, animal and plant components, fire frequency, and changes in soil properties, as well as the reduction of landscape diversity (Rey Benayas, 2007; de Araújo et al., 2015; Keesstra et al., 2016).

In particular, the abandonment of hinland rural areas has led to a decrease in biodiversity associated with Mediterranean landscapes (Rippa et al., 2011; Troiano et al., 2021) due to an increase in forests and scrubland (Evangelista et al., 2016) and reforestation dynamics (Vacchiano et al., 2017).

The introduction/reintroduction of animal species (*e.g.*, large herbivores), a process also named rewilding, may contribute to restoring the natural processes (Svenning et al., 2016). Rewilding has several definitions, which have different temporal references and geographic applicability (Jørgensen, 2015), such as Pleistocene rewilding, ecological rewilding, passive rewilding, and trophic rewilding (Corlett, 2016). The latter, in particular, seems to fit best in our case study, where wild herbivores are potentially shown to be a rewilding tool (Van Klink et al., 2020) due to their important bottom-up and top-down pressures in structuring ecological communities (Martin et al., 2010).

Here, we provide data to support the claim that the reintroduction of large herbivores may help maintain the open habitats (Gordon et al., 2021; Kowalczyk, Kamiński & Borowik, 2021), contributing to landscape biodiversity. In fact, large herbivores promote landscape-scale biodiversity by creating environmental heterogeneity (through browsing and grazing) and through the dispersion of propagules (Svenning, 2020). They can also exert non-trophic impacts through trampling action (Stavi et al., 2009), which results in the tearing of the vegetation and the heterogeneous soil compaction (Stavi et al., 2021).

There are several examples of wild herbivore reintroductions in Europe, including the reintroduction of bison (*Bison bonasus*) in the Romanian Carpathians (Vasile, 2018), the introduction of wild horse (*Equus ferus*) in Sweden (Garrido et al., 2021), the reintroduction of roe deer (*Capreolus capreolus*) (Calenge et al., 2005) and red deer (*Cervus elaphus*) in Corsica (Kidjo et al., 2007). In particular, no study has ever addressed the cumulative effect on vegetation structure of these wild deer grazers, also combined with domestic grazing.

The goal of our work is to evaluate the effect of the reintroduction and spreading of wild deer, combined to livestock in grazing in open areas (grassland).

Our research represents a valuable opportunity to explore the use of these species as potential landscapes management tool, to moderate the impact of farmland abandonment and the subsequent forest encroachment. It could also an opportunity to conserve the *C. c. italicus*, an Italian endemic subspecies assessed as Vulnerable (VU-D2) by the International Union for Conservation of Nature (Rondinini et al., 2013).

## Materials & Methods

### Study area

**Figure 1:**
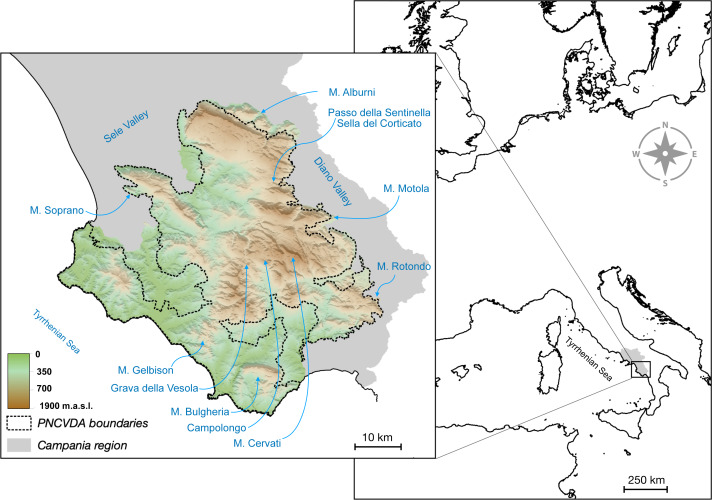
Study area. Topography of the Cilento region with the administrative boundaries and the toponyms considered in the study.

Our study was conducted in the Cilento region (40°12′ N−15°12′E, in the Southern Apennines), defined as the Cilento, Vallo di Diano e Alburni National Park (PNCVDA) and its immediate surroundings (Fig. 1). The PNCVDA is the largest protected area in Italy, including marine, lowlands and mountainous areas. In particular, the latter are characterized by a Mediterranean landscape, structured as a mosaic of woody, natural and agricultural patches, with small rural areas.

At high elevations (with Mount Cervati reaching 1,898 m a.s.l.) the habitat is dominated by beech (*Fagus sylvatica*) forests, interspersed with secondary grasslands (*sensus* Dengler et al., 2020) originated in historical time (Blondel, 2010). These are high nature value grasslands (Veen et al., 2009; Oppermann, Beaufoy & Jones, 2012; Török & Dengler, 2018), where the natural conditions are more or less unaltered, sometimes after cessation of restation, fire, herbivory and human arable fields (Dengler et al., 2020). At lower elevations, forests of *Alnus cordata* and *Castanea sativa* dominate, followed by deciduous oaks (*Quercus cerris*, *Quercus pubescens*) and maple trees (*Acer* sp.). Nowadays, these forests are populated mostly by Italian hare (endemism *Lepus corsicanus*; Buglione et al., 2018), wild boar (Fulgione et al., 2016; Maselli et al., 2016; Maselli et al., 2014), roe deer, red deer and by the wolf (*Canis lupus*), the main predator in the region, expanding at a fast pace with a population currently estimated at many dozens of individuals (Buglione et al., 2020a; Fulgione & Buglione, 2022).

The study area, since the 1950s, is strongly affected by farmland abandonment (Bakudila et al., 2015) and by changes in traditional practices (de Filippo, de Luca & Nicoletti, 2002), so named non-mechanized, non-intensive or extensive agriculture (Hamadani et al., 2021) with pastures in fragmented patches. This phenomenon has determine a loss of biodiversity (Cuttelod et al., 2009; Underwood et al., 2009), primarily as a consequence of the loss endemism and those species strongly linked to the human activity (*e.g.*, *Emberiza cirlus*, *Alectoris graeca, Antus trivialis;* (Rippa et al., 2011; Brambilla et al., 2010; Brambilla, 2015; Regos et al., 2016)). In addiction, the absence of large grazing animals determined a competition between plant species for light, inducing dominances resulting in decline of diversity of plant species in grasslands (Allan, 2022). Finally, the abandonment of agricultural areas also involves a loss of the typical Mediterranean patches, resulting in a uniformity of the landscape and a reduction of edge population (Buglione et al., 2020b; Mantero et al., 2020).

The abandonment can be considered as the main driver of landscape change given that in the PNCVDA the human activity in the forest (such as logging, construction of roads, fences or houses) are limited and under rules.

In this area, roe deer and red deer were extinct, dating back to the early 1950s, as a consequence of several factors including human persecution and habitat changes. However, roe deer and red deer were reintroduced between 2003–2006 (37 roe deer released in the Campolongo locality and 35 red deer released in the Grava della Vesola locality) (Fig. 1).

### Analysis of population

In order to simulate the demography of expansion of the wild ungulates, we performed a population viability analysis (PVA). The PVA was largely adopted for evaluation of management action in endangered species, using even the supplementation scenario to examine the relative benefits of alternative management actions (Ralls, Beissinger & Cochrane, 2002; Lacy, 1993; Burgman, 2005). In reintroduced population, PVA is used to estimate, even in a short period, the mean generation and future demographic trajectory (Vonholdt et al., 2008; South, Rushton & Macdonald, 2000; Armstrong & Ewen, 2002). It is a tool to modulate reintroduction design, future reintroductions and population management.

The PVA was performed in Vortex 10.1.6.0 software (Lacy, Miller & Traylor-Holzer, 2015), putting biological parameters and ecological data for roe deer (Flajšman et al., 2018; Hewison et al., 1999; Hewison & Gaillard, 2001; Mauget, Mauget & Sempéré, 2003; Vanpé et al., 2008), as well as for red deer (Albon et al., 2008; Borowik, Wawrzyniak & Jędrzejewska, 2016; Clutton-Brock, Major & Guinness, 1985; Krzywiński, 1981; Stewart et al., 2005). For both demographic simulations, with 100 iterations each, we compared the number of individuals living currently in the study area with the number expected to be found in 16–20 years since their reintroduction turning on the option “the extinction threshold for the disappearance of each individual of one of the two sexes”.

### Habitat suitability analysis

To collected data for spatial elaborations, we gathered direct and indirect (*i.e.*, footprints, excrement, barking of trees, shrub browsing) presence information ascribable to the roe deer and red deer, by using diurnal and nocturnal surveys, with walking transects and spotlight method (Parkes, 2001).

To define the potential distribution for roe deer and red deer, presence data were employed in spatial elaboration by using Maximum Entropy Distribution Model (MaxEnt, Phillips, Anderson & Schapire, 2006) in MaxEnt 3.4.1 software (http://www.cs.princeton.edu/).

A total of 13 spatially environmental variables were selected: elevation, aspect, slope, roads, waterways, broadleaf forests, mixed woods, conifer forests, scrublands, grasslands, tree plantations, agricultural fields, urban area.

Elevation was obtained as a Digital Terrain Model (DTM) from the National Geoportal (http://www.pcn.minambiente.it/mattm/), while both aspect and slope were calculated from the DTM, using “Aspect” and “Slope” GDAL functions in QGIS 3.10 (https://www.qgis.org). The remaining variables were obtained as categorical vector files from Corine Land Cover 2018 classes (EEA, 2018). Then, in the model we considered the Euclidean distance from each point of presence to each categorical variable. To do this, the GDAL function Proximity (raster distance) was implemented in QGIS 3.10, by setting distance units to “geographical units”.

Pearson’s correlation coefficient was calculated to avoid collinearity between the predictive variables and, after the test, distance from urban areas variable was excluded from analysis.

MaxEnt model settings that best fit our data were selected using the R package “ENMeval” (Muscarella et al., 2014). The software implements features of five classes (linear, quadratic, product, threshold, and hinge) and the following alternative combinations of features were tested: linear, linear + quadratic, hinge, linear + quadratic + hinge, linear + quadratic + hinge + product and linear + quadratic + hinge + product + threshold (Muscarella et al., 2014), as well as the regularization values between 0.5 and 4, with 0.5 increments. The model with the lowest Akaike Information Criterion (AICc) was chosen out of the 48 combinations, corrected for a small sample size (Warren & Seifert, 2011). Predictive performance was evaluated by calculating the area under the receiver operating characteristic curve (AUC) (Swets, 1988).

The habitat suitability maps was obtained converting habitat suitable area in discrete maps using “10th percentile training presence” (Pearson et al., 2006) and “maximum training sensitivity plus specificity” as thresholds (Liu, Newell & White, 2016). The total area included within these thresholds was used as suitable habitat area and constituted the clipping mask in future analyses.

The evaluation of suitable area for the two species was also useful to select where to install the camera traps.

### Animal distribution and density

To estimate distribution and density of roe deer, red deer and livestock, we used data from camera trapping, that has been proven to be a reliable method to obtain density estimation of wild and livestock populations, compared to other more expensive and time-consuming techniques (Palencia et al., 2021; Romani et al., 2018).

**Figure 2:**
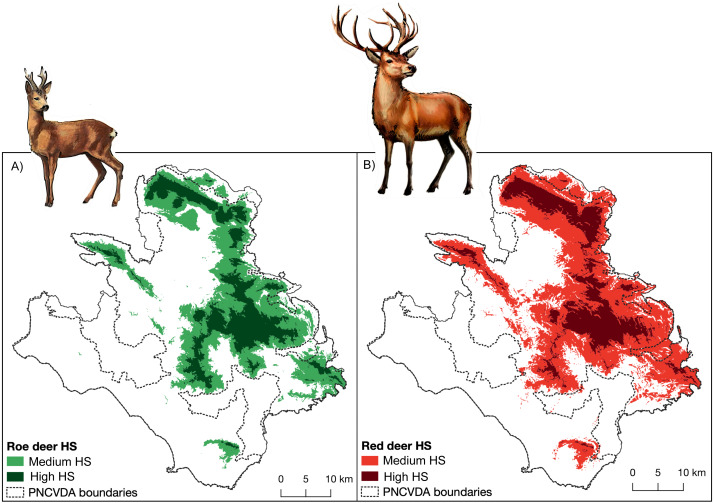
Habitat suitability. Habitat suitability maps obtained from SDMs, (A) for roe deer and (B) for red deer.

Locations for cameras were selected by dividing the study area in 10 × 10 km UTM quadrants and selecting 12 quadrants which included suitable habitat for roe deer and red deer, gradually distant from the known point of reintroduction of the species (Fig. 2). Within each quadrant, one smaller cell of 2 × 2 km was selected randomly, and four camera traps were placed as close as possible to the four corners of each cell, based on feasibly reachable locations (Tables S1 and S2).

All cameras had a detection radius (r) = 20 m, except for Uovision UV575, (*r* = 12 m). Detection zone and radius were obtained from manufacturer manuals for each camera trap model used (according to Rowcliffe et al., 2008). Detections were considered independent if they were at least ≥ 10 min apart.

Camera traps were affixed to trees, around 1.50 m from the ground, pointed in the direction most likely to record animal activity and preferentially oriented in order to minimize sun glares. The devices were set to video mode, with a recording length of 30 s and with a refractory period varying from one to ten minutes. PIR sensitivity was set to normal in locations with open vegetation, and to low in locations with surrounding vegetation, to reduce the risk of accidental camera activations due to movement of branches and/or leaves. The camera traps were left active for an average of 15 days, before being moved to a different cell.

In analyzing the video trapping data, we evaluated every sign that allowed to distinguish individuals, such as the horns shape, marks of predation or fighting, peculiarities of the coat color.

Camera trapping took place from spring to fall 2021 and was carried out using: 8 Scout Guard SG-2060-X cameras, with a detection zone *θ* = 0.87 rad, 4 LTL Acorn 5310 with a detection zone *θ* = 2.09 rad, 2 Browning Recon Force 4K with a detection zone *θ* = 0.77 rad, 2 Stealth Cam DS4K, with a detection zone *θ* = 0.79 rad, 2 Victure HC300, with a detection zone *θ* = 1.57 rad, 2 Victure HC520, with a detection zone *θ* = 1.57 rad, and 4 Uovision UV575, with a detection zone *θ* = 1.75 rad. Density of roe deer, red deer and livestock was estimated according to the Random Encounter Model (REM) (Rowcliffe et al., 2008) in R (R Core Team, 2020), using “camtools” functions (Palencia et al., 2021), taking into account the camera trap models, with the following equation:

\begin{eqnarray*}D= \frac{y}{t} \frac{\pi }{vr \left( 2+\theta \right) } \end{eqnarray*}

where D represents the density (individuals/km^2^), *y*/*t* is the trapping rate (expressed as the ratio of independent capture events - *y* - by the number of trap days - *t* -, times 100), r (radius of the detection zone) and *θ* (the angle of the detection zone) are the camera trap detection parameters, *v* represents the animal movement speed (Rowcliffe et al., 2008).

When different models of cameras have been used for the same quadrant, radius and angle of detection zone were average. Averaged daily movements for ungulate species and livestock were obtained from the literature: 1.99 km/day for roe deer (Pépin et al., 2004; Romani et al., 2018), and 2.88 km/day for red deer (Pépin et al., 2004; Pépin, Morellet & Goulard, 2009). Average daily movements for livestock were 4 km/day for cattle (Pauler et al., 2020), 6 km/day for sheep (Broom, 2021), and 7.2 km/day for horses (Hampson et al., 2010).

For each species, we estimated the average density for the study area as well as local densities for each surveyed cell, along gradually distant areas from the release locations. The density of each cell was interpolated using the IWD Interpolation feature in QGIS and clipped to the extent of suitable habitat for each species. To obtain the distribution of density, we fitted GLM models (using Pseudo-R^2^) selecting an “Inverse gaussian” family for roe deer and “Gamma” family for red deer, using the R package “lme4”. We considered density as a response variable, and distance from release points as a predictor variable. The latter was calculated using the algorithm Proximity (raster distance) implemented in QGIS 3.10, by setting distance units to “geographical units”. This allowed also to understand whether our deer populations are represented exclusively by the reintroduced individuals or have been enriched by other. Finally, to obtain the area affected by the cumulative presence of grazers, the livestock density was merged to roe deer and red deer density rasters.

### Landscape changes

To infer the potential effect of reintroduced wild herbivores and livestock on the landscape, we compared the land use and land cover from 2000 and 2018, considering Level III Corine Land Cover (Bossard, Feranec & Otahel, 2000; EEA, 2018), a third-level hierarchical classification system with 44 classes (*i.e.*, artificial surfaces, agricultural areas, forest and semi natural areas), in a scale of 1:100,000, with a minimum cartographic unit (MCU) of 25 ha, a geometric accuracy better than 100 m.

To obtain areas where land use changes occurred, we compared vector layers for CLC classification from 2000 and 2018 using the *overlay* function in QGIS Layers, cleaning polygons smaller than 0.25 ha, as these are generally misalignment artifacts or represent areas too small to have a significant impact on the landscape (Büttner & Kosztra, 2011). The resulting polygons were categorized in different land use change classes according to the type of change (de Filippo, de Luca & Nicoletti, 2002). We considered *Spontaneous* and *Inverse successions*, as they are opposing processes and are strongly linked to herbivore activity.

*Spontaneous Succession* (*SS*), indicator of loss of semi-open habitats (open area surrounded by the forest), was defined as the transformation of natural areas (class 3) into more advanced succession stages according to natural dynamics (grasslands in bushes, bushes in woods, *etc*.).

*Inverse Succession* (*IS*), indicator of landscape opening areas, was defined as transition from natural advanced forms to less advanced states of succession, for example from woods to bushes (*e.g.*, 311 in 322 or 321 or 312 in 322 or 323).

The polygons were subsequently rasterized to obtain files comparable with the density distributions (100 × 100 m resolution).

To infer on the combined effect of wild and domestic grazers’ pressure, we compared the mean grazing density in areas where *Inverse* and *Spontaneous succession* occurred, as well as where they not occurred. We tested the differences using Welch’s *t*-test, to account for different sample sizes as the number of pixels where the changes occurred different.

## Results

The PVA showed that in 16–19 years, the population size of roe deer expanded from 37 released individuals to an estimated population of 175.61 (SE = 9.24) while the red deer expanded from 35 to 84.28 (SE = 9.95).

For roe deer, the most suitable settings for the habitat suitability model are linear + quadratic features with a regularization multiplier of four; while for red deer, linear features with a regularization multiplier of three. The test AUC for the final models is 0.880 and 0.862 for roe deer and red deer, respectively. The 10th percentile training presence threshold results in values of 0.221 for roe deer and 0.209 for red deer, while the maximum training sensitivity plus specificity threshold results in values of 0.559 for roe deer and 0.621 for red deer.

The highly suitable territories for the two species are closely associated with mountainous areas (Fig. 2), in which elevation is the strongest predictive variable, explaining the 71.7% and 70.5% of the variation for roe deer and red deer, respectively (Table 1). Areas include two mountain groups, the Alburni Mountains and Cervati/Motola Mountains, connected by an ecological corridor running through Sella del Corticato and Passo della Sentinella, in the North-South direction. These areas overlap with the distribution of wolves (Buglione et al., 2020a) which also include the Soprano Mountain, the Bulgheria Mountain, Gelbison Mountain and Rotondo Mountain, although the latter area less connected (Fig. 2 cfr Fig. 1).

**Table 1:** Environmental variables. Estimates of relative contributions of the environmental variables to the Maxent models.

	Contribution (%)	
Variables	Roe deer	Red deer
Elevation	71.7	70.5
Agricultural meadows	10.7	14.9
Tree plantations	9.0	0.1
Waterways	3.5	0.0
Grasslands	1.9	5.6
Slope	1.3	5.8
Mixed woods	1.1	1.7
Aspect	0.5	0.0
Broadleaves	0.2	0.0
Roads	0.1	1.3
Conifers	0.1	0.0
Scrub	0.0	0.0

Analyses of the contributions of the variables show that the second relevant environmental variable affecting habitat suitability is the distance from agricultural meadows (10.7% and 14.9% for roe deer and red deer respectively), followed by the tree plantations distance for roe deer (9%), and by the slope (5.8%) and grasslands distance (5.6%) for red deer. All other variables show a contribution equal to or less than 5% (Table 1).

The suitable area for roe deer and red deer extends for about 687 km^2^ and 880 km^2^, respectively (Figs. 2C, 2D). Considering the different levels of suitability, greater extension of the medium level in roe deer is observed (454 km^2^). Conversely, in red deer the largest extension is observed for highly level of suitability (642 km^2^) (Table 2). A spatial overlap between the high suitability for the two species exists for many local areas (for an extent of 187 km^2^), reaching a value of 665 km^2^ considering the total suitable area.

Furthermore, considering the population size estimated by PVA and highly suitable habitat (assuming a more parsimonious approach), we estimated about 0.75 ind/km^2^ of roe deer and 0.13 ind/km^2^ of red deer.

**Figure 3:**
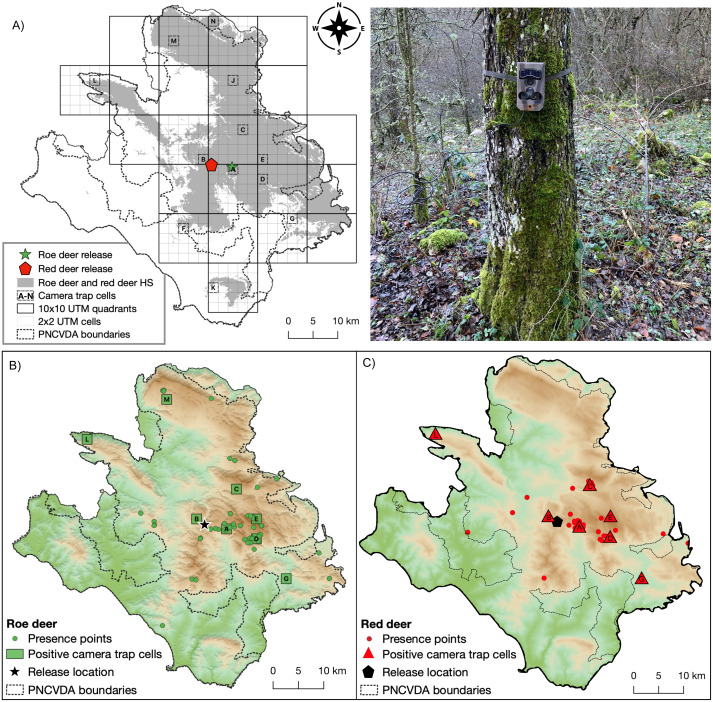
Camera trap locations and Species occurrences. (A) Sampling cells (from A to N) included in 10 × 10 km quadrants encompassing suitable habitat for both wild ungulates; on the right a typical camera trap installation site. Presence points (dots) and camera trap cells (lettered squares) where (B) roe deer and (C) red deer occurrences were detected.

In total, 12 cells (with four cameras each) were surveyed, for 2876 trapping days (Fig. 3A). Roe deer presence is detected in eight cells (Fig. 3B), red deer in seven cells (Fig. 3C), and the livestock in 10 cells.

**Table 2:** Habitat suitability. Extension of habitat (km^2^) for roe deer and red deer, merged in two levels of suitability.

	Roe deer	Red deer
Total	687	880
Medium	454	238
Highly	233	642

The current mean densities for roe deer are three times as much (1.57 ind/km^2^, SE = 0.37) as those estimated for red deer (0.54 ind/km^2^, SE = 0.25).

**Figure 4:**
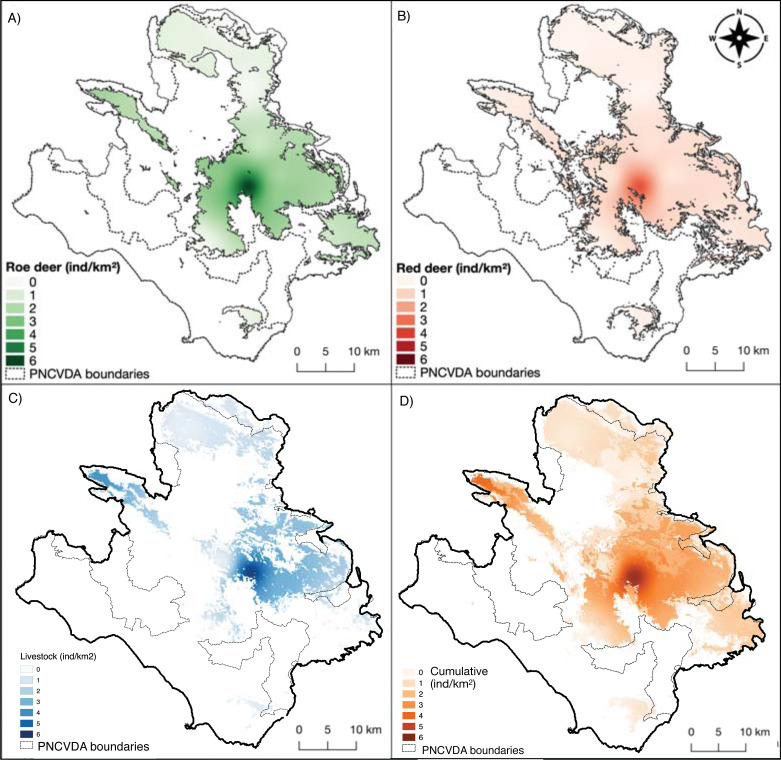
Density maps. (A) Roe deer and (B) red deer densities clipped within their respective suitable habitats; (C) livestock density; (D) cumulative density.

The highest density obtained for these two wild ungulates is close to their release point (6.01 ± 1.48 and 3.56 ± 2.44 mean ind/km^2^ ± SE for roe and red deer, respectively) (Figs. 4A, 4B), and these values decrease moving away from this area (Table 3). Distance from release points was a good predictor to explain the density distribution, with models’ Pseudo-R^2^ of 0.53 for roe deer and 0.84 for red deer (Table 4), with a *p*-value <0.0001 for both.

**Table 3:** Animal densities. Roe deer, red deer and livestock densities (mean ind/km^2^ ± SE) of each sampling cells.

Cell	Roe deer	Red deer	Livestock
A	6.01 ± 1.48	3.56 ± 2.44	4.14 ± 0.82
B	2.60 ± 1.12	0.51 ± 0.21	0.62 ± 0.11
C	1.32 ± 0.56	0.59 ± 0.15	1.69 ± 0.44
D	2.48 ± 0.47	0.86 ± 0.42	3.72 ± 0.97
E	2.11 ± 0.88	0.42 ± 0.15	0.00 ± 0.00
F	0.00 ± 0.00	0.00 ± 0.00	0.20 ± 0.06
G	1.17 ± 1.02	0.27 ± 0.10	0.25 ± 0.06
J	0.00 ± 0.00	0.00 ± 0.00	0.00 ± 0.00
K	0.00 ± 0.00	0.00 ± 0.00	0.20 ± 0.05
L	2.25 ± 1.59	0.22 ± 0.08	2.57 ± 0.67
M	0.55 ± 0.19	0.00 ± 0.00	0.36 ± 0.04
N	0.00 ± 0.00	0.00 ± 0.00	0.67 ± 0.17

**Table 4:** GLM. GLM results for distance from release points as a predictor variable for the densities of roe deer and red deer (N= Occurrences, BIC = Bayesian Information Criterion, AIC = Akaike Information Criterion).

	Roe deer	Red deer
Intercept	1.68 (*p* < 0.001)	2.43 (*p* < 0.001)
Distance from release points	−0.79 (*p* < 0.001)	1.18 (*p* < 0.001)
N	68634	87889
AIC	151036.61	20614.93
BIC	151064.02	20643.08
Pseudo-R^2^	0.57	0.84

The deer share the pasture hosting a livestock mean density of 1.78 ind/km^2^ (SE = 0.60) (Fig. 4C). The density of livestock, is highest both close to the release point of the deer and around M. Soprano and M. Cervati.

The average cumulative density of all grazers is equal to 1.51 ind/km^2^ (SE = 0.02), and overlaps with high suitability of wild grazers, with the exception for the Northern portion of the study area (Alburni Mountains) (Fig. 4D).

**Figure 5:**
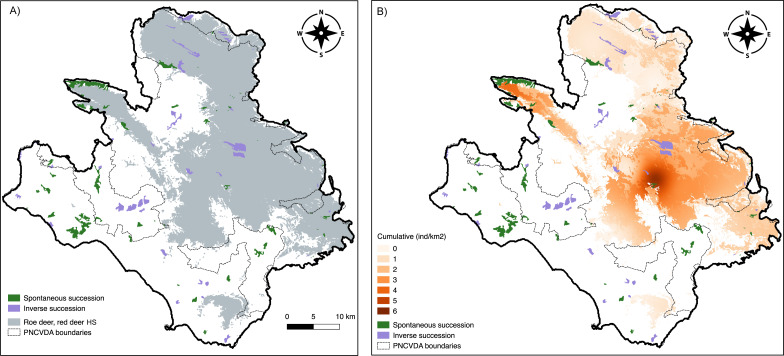
Land use change. *Spontaneous* and *Inverse Succession* in relation to (A) suitable habitat for roe deer and red deer and (B) the cumulative density of deer and livestock.

In total, 26.30 km^2^ of *Inverse Succession* and 32.26 km^2^ of *Spontaneous Succession* are found in the study area (Fig. 5). Of these, 11.71 km^2^ (*IS*) and 4.33 km^2^ (*SS*) are included in the suitable area for roe deer and red deer, whereas 14.59 km^2^ and 27.93 km^2^ are outside of the suitable habitat for these ungulates (Fig. 5A). A chi-squared test on the distribution of *Inverse* and *Spontaneous Succession* sites within and outside this area revealed a statistical difference *X* ^2^ (1, *N* = 5856) = 2,175, *p* < 0.0001, indicating a significantly higher frequency of *Spontaneous Succession* outside the suitable range for the ungulates.

Cumulative density of ungulates and livestock in sites classified as *Spontaneous Succession* was significantly lower compared to density in *Inverse Succession* (Fig. 5B). The sites where a *Spontaneous Succession* is observed are well used by 2.41 ind/km^2^ (SE = 0.14) of all ungulates. Instead, the *Inverse Succession* sites are home of 11.92 ind/km^2^ (SE = 0.10) grazers. Differences between densities in *SS* and *IS* are significant using Welch’s *t*-test (*p* < 0.01).

**Figure 6:**
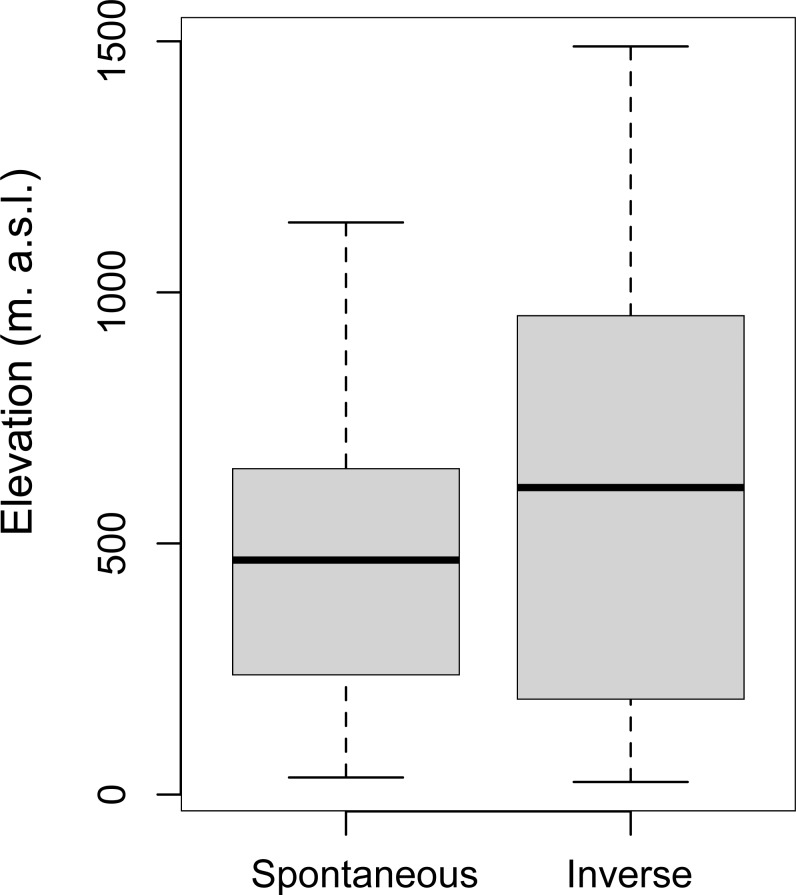
Correlation between elevation and *Successions*. Variation of elevation (meters above sea level, m. a.s.l.) in *Spontaneous* and *Inverse Succession* areas without roe deer and red deer.

Since elevation is one of the dominant factors in driving the dynamics of the vegetational succession (Gillet, Besson & Gobat, 2002), To rule out that elevation was responsible for the landscape changes, we have analyzed this contribution by correlating the *Spontaneous Succession* of patches not inhabited by deer to their altitudinal levels. There was no significant difference in elevation between the areas with *Spontaneous Succession* (*M* = 473.27; SD = 275.03) and *Inverse Succession* (*M* = 611.59; SD = 416.67; (*t*-test, *p* < 0.05) (Fig. 6).

## Discussion

The population analysis of wild ungulates, formerly extinct in historical times and then reintroduced, has clearly shown a good capacity of expansion and growth.

The demography of the deer has reached remarkable population size if we consider the data obtained both from the population growth model (PVA) and through the survey data. This increase depends exclusively on the reintroduced individuals without any immigrants from surrounded areas, based on the observed spatial pattern of the populations. Moreover, this must also be considered in light of the growing of wolf population in the last decade (Buglione et al., 2020a), probably, focused primarily on the more abundant and well adapted wild boar (Fulgione et al., 2016; Fulgione et al., 2017). However, it is a promising result for *C. c. italicus* that opens motivating conservation implications for this Italian endemism.

Interestingly, the suitable habitat for these wild ungulates is all placed in the central portion of the study area whereas the density is decreasing in the boundaries. The most possible interpretation of this observation could be that Cilento behaves like an isolated land that disconnected from other suitable areas due to the marine coastline in the south and west, and the two large anthropized valleys in the north and east (Valle del Sele and Vallo di Diano, Fig. 1).

It is difficult to predict the trajectory of the current population, however, a valuable indication is given by the distribution of the suitable habitat and the spatial variations of density recorded in it. Indeed, Cilento roe deer population and the Southern Apennines main population can overlap their territory in the near future or they could persist as isolated as they appear now.

The reintroduction is considered not only a conservation action but also a useful management tool for landscape modeling. However, it need to take in mind deer ecology, landscape historical evolution, as well as supplementary release to ensure long-term persistence of the population.

After the extinction of roe deer and red deer, the forest encroachment was probably braked by an intense use of the mountain by humans. Nevertheless, still the traditional practices are ongoing in these areas with a higher density of grazing domestic animals. Following the abandonment of the mountains, this grazing was lowered triggering an uniformization of the landscape with a gradual regained of the forest (Vacchiano et al., 2017) and loss of biodiversity linked to farmlands and human activities (Troiano et al., 2021).

Considering the cumulative impact of domestic and wild grazers, we noticed interesting outcomes on the landscape. The areas in which we have recorded a more pronounced increase of the *Spontaneous Succession* is correlated with a lower density of grazers. These results suggest that the wild grazers prefer semi-open areas and, in association with domestic grazing, they could be one of the determining elements in retarding the advancement of the forest, maintaining the landscape mosaic of the Apennine Mountains.

However, it is essential to demonstrate cause-and-effect relationships: are the ungulates controlling the *Succession* with their presence or are the areas with *Inverse Succession* more suitable for these species? The non-correlation between the elevation and the *Successions* in the patches not housing deer, is a useful preliminary indication for excluding the effect of this variable in the vegetation dynamics. However, it is still necessary to develop more comprehensive studies including also the effect of climate variables linked with the long-term vegetation dynamics.

Our data have supported the hypothesis that the introduction *ad hoc* of wild ungulates may be useful for the maintenance of open habitats. The relative time in which these results can be achieved should not underestimate the use of this tool. The movement of wild animals and the reintroduction into new communities that have found their equilibrium is always a very complicated operation, because it collides with health, population genetic, ecosystem, and human dimension implications.

## Conclusions

The reintroduction of wild ungulates to mountainous Apennine, combined with the already present livestock, retard the advancement of the forest and could represent a very suitable tool both to improve diversity in animal communities, shaping vegetation and landscape. Furthermore, this strategy shows a potential as conservation tool to sustain an endemic population of roe deer deserving of protection.

##  Supplemental Information

10.7717/peerj.14492/supp-1 Supplemental Information 1 Coordinates of presence points for investigated animals Coordinates of presence point for *Capreolus capreolus*, *Cervus elaphus* and livestock (cattle, sheep and horses), WGS 84/UTM zone 33N reference system.

10.7717/peerj.14492/supp-2 Supplemental Information 2 Coordinate of sampled cell Cell coordinates in which species density was estimated, WGS 84/UTM zone 33N reference system.

## Additional Information and Declarations

### Data Availability

The following information was supplied regarding data availability: The raw data is available in the Supplementary Files.
